# Supplementary material for: Peroxiredoxin 2 is required for the redox mediated adaptation to exercise
Source: Redox Biol. 2023 Feb 9;60:102631. doi: 10.1016/j.redox.2023.102631 (PMC9950660; doi:10.1016/j.redox.2023.102631)
Supplement: Multimedia component 1 [file mmc1.docx]

**Supplementary Figure 1**. Effects of different concentrations of H_2_O_2_ on monomer/dimer ratio analysed by immunoblotting for Prdx3 (a), Prdx5 (b), and Prdx6 (c) in C2C12 myoblasts 25 µM H_2_O_2_ for 10 min. Immunoblotting for monomer/dimer of PRDX1 (d) and PRDX2 (e) and Prdx SO_2_/SO_3_ (f) following 25 µM H_2_O_2_ treatment for 1, 5, 10, 40 or 60 min. Immunoblotting of proteins involved in mitochondrial turnover (Protein DJ-1, Parkin and Bnip3) in C2C12 myoblasts treated with 25 µM H_2_O_2_ for 10 min and allowed to proliferate for 3h and 24h. Graphs are the mean +/- SEM and all experiments were performed with at least n=3-6, one-way ANOVA was used for significance between groups and *p-*value of <0.05 was considered as statistically significant *(*p*<0.05). *p values (d: Ctrl vs 10 mins= 0.0248; e: Ctrl vs 10 mins= 0.0003; f: Ctrl vs 10 mins< 0.0001; g: Ctrl vs 24h= 0.0117; h: Ctrl vs 24h= 0.0495; i: Ctrl vs 24h< 0.0001).*

**Supplementary Figure 2**. Schematic of approach for proliferation and differentiation of C2C12 cells following 10 min with 25 µM H_2_O_2_ (a). Western blot analysis expression during the proliferation and differentiation of C2C12s controls and cells treated for 10 min with 25 µM H_2_O_2_ for TRX2, Parkin, p62 and LC3 II/I (b-e). Cells with siPrdx1 and/or Prdx2 and treated with H_2_O_2_ and Western blot analysis for SOD2, p62 and Parkin (f-h). C2C12 cells with nuclear localisation of NRF2 and STAT3 following siPrdx1 and/or Prdx2 and treatment with H_2_O_2_, scale bar =75µm. Graphs are the mean +/- SEM and all experiments with n=3, two-way ANOVA analysis was used between groups and *p-*value of <0.05 was considered as statistically significant *(*p*<0.05). (a-k). *p values (b: Ctrl: PM1 vs Ctrl: D5= 0.0050, Ctrl: PM2 vs H2O2: PM2= 0.0330; c: Ctrl: PM1 vs Ctrl: D5= 0.0470, Ctrl: PM2 vs H2O2: PM2= 0.0084; d: Ctrl: PM2 vs Ctrl: D5= 0.0017, Ctrl: PM2 vs H2O2: PM2= 0.0011; e: Ctrl: PM1 vs Ctrl: D5< 0.0001; f: Ctrl: Ctrl vs H2O2: Ctrl= 0.0005; g: Ctrl: Ctrl vs Ctrl: siPrdx2= 0.0453, Ctrl: Ctrl vs H2O2: Ctrl= 0.0314; h: Ctrl: Ctrl vs H2O2: Ctrl= 0.0370; i: Ctrl: Ctrl vs H2O2: Ctrl< 0.0001; j: Ctrl: Ctrl vs H2O2: Ctrl= 0.0003)*.

**Supplementary Figure 3**. (a) Representative images of DCFDA staining following 90 mins of exercise protocol, data is represented as relative fluorescence intensity and experiments performed with at least 60 worms, scale bar = 275µm. Immunoblotting for PRDX-2 expression in N2, *prdx-2* (b) and *skn-1* (c) strains following a 5day exercise protocol. Non-reducing immunoblot for analysis of monomer/dimer formation of PRDX-2 in N2, *prdx-2* (d) and *skn-1* (e) strains following 5 day exercise protocol. Body size of N2, *prdx-2 (gk163)* and *skn-1 (zj15)* strains, experiments were performed with at least 45 worms per experiment (f). Graphs are the mean +/- SEM, all experiments were performed n=3-4 and analysed by Student *t* test and *p-*value of <0.05 was considered as statistically significant *(*p*<0.05). *p values (a: Control vs Exercise= 0.0324; b: N2: Control vs prdx-2 (gk163): Control< 0.0001; c: Control vs Exercise= 0.0420; f: Body size: N2: Control vs Exercise= 0.0340, prdx-2 (gk163): Control vs Exercise= 0.0107, skn-1 (zj15): Control vs Exercise= 0.0612).*

**Supplementary Figure 4**. Schematic diagram of the redox proteomic approach to quantify protein abundance by label free quantification and relative quantification of the reversible oxidation state of peptides containing redox sensitive Cys residues.

**Supplementary Table 1**. List of Cys containing peptides of where the redox state is significantly changed comparing the Log_2_Fold change of ratio of Heavy NEM:NEM labelling of Cys residues in non-exercised strains N2 Vs *prdx-2* and N2 Vs *skn-1* and following exercise compared to non-exercised controls in N2, *prdx-2* and *skn-1* strains. Peptides coloured blue are Cys residues more reduced and those in orange are more oxidised, * have p-value <0.05.

**Supplementary Table 2**. Data from longevity and survival assays related to Figure 4. The figures the data are related to are included in the table.

**Supplementary File 1**. Label free quantification proteomic data from N2, *prdx-2*, *skn-1* and *bli-3* strains with and without swimming exercise including differential analysis of proteins.

**Supplementary File 2**. Redox proteomic analysis of reversible oxidation state of Cys containing peptides labelled with both light and heavy NEM, Log_2_FoldChange and p-value.

**Suppl. Fig1**


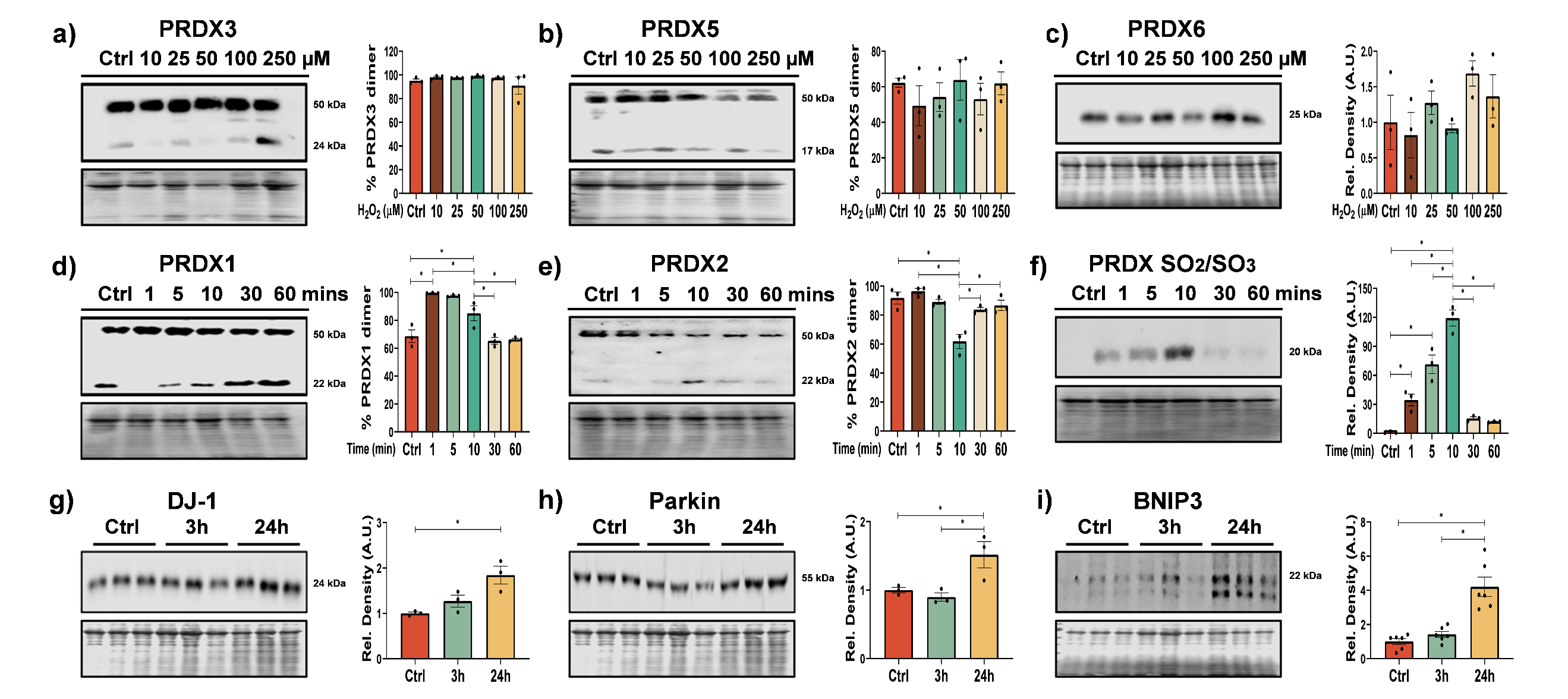


**Suppl. Fig2**


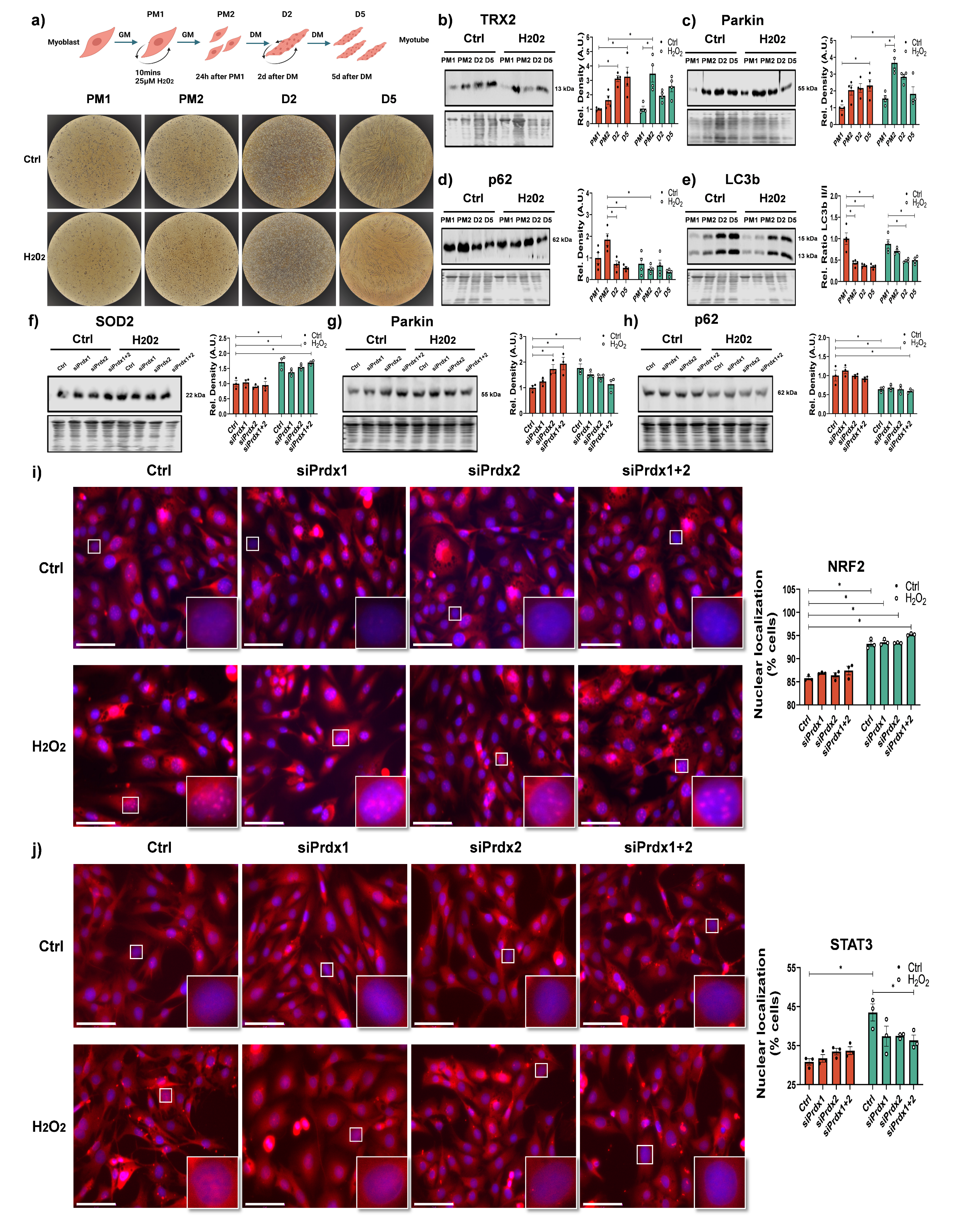


**Suppl. Fig3**


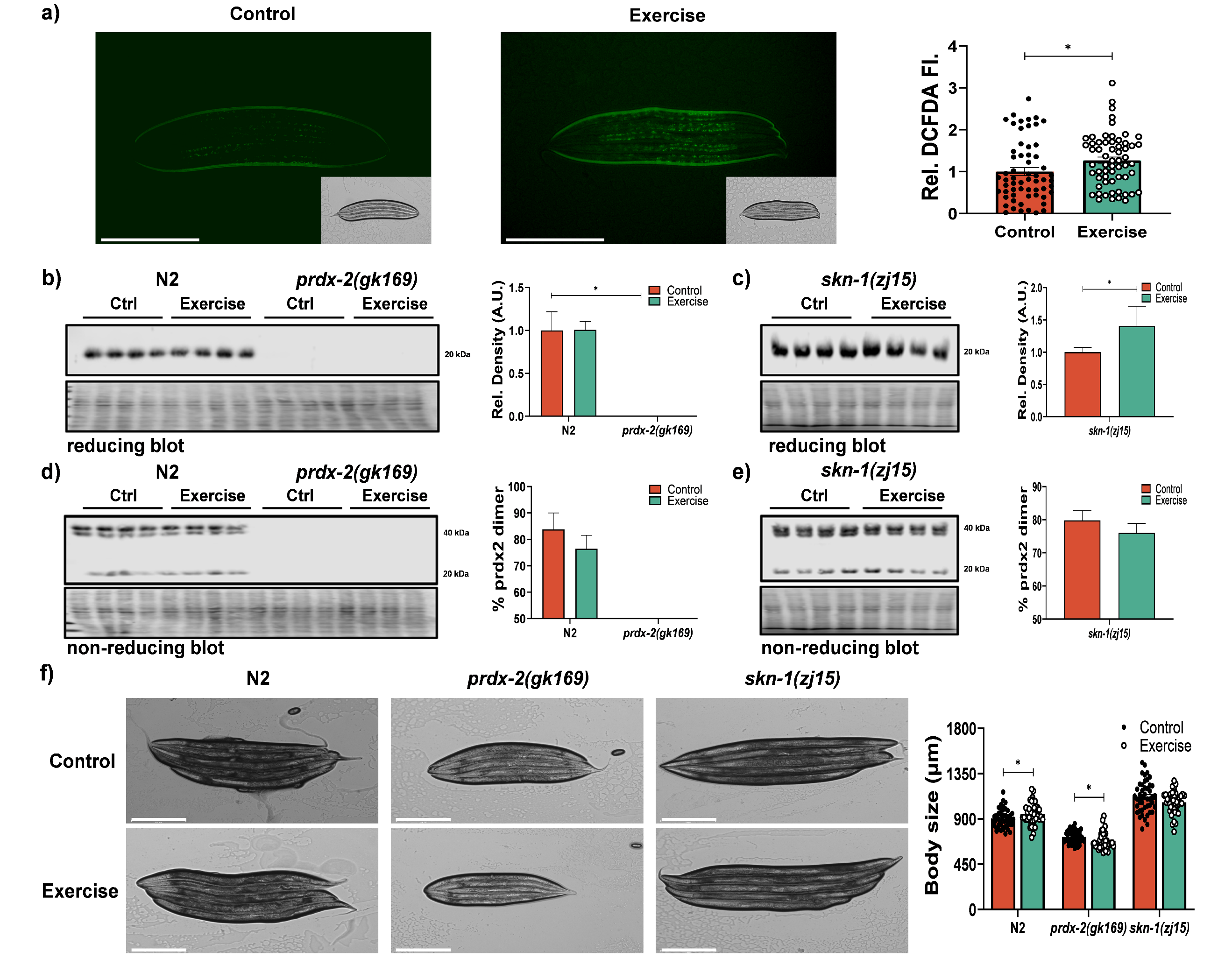


**Suppl. Fig 4**


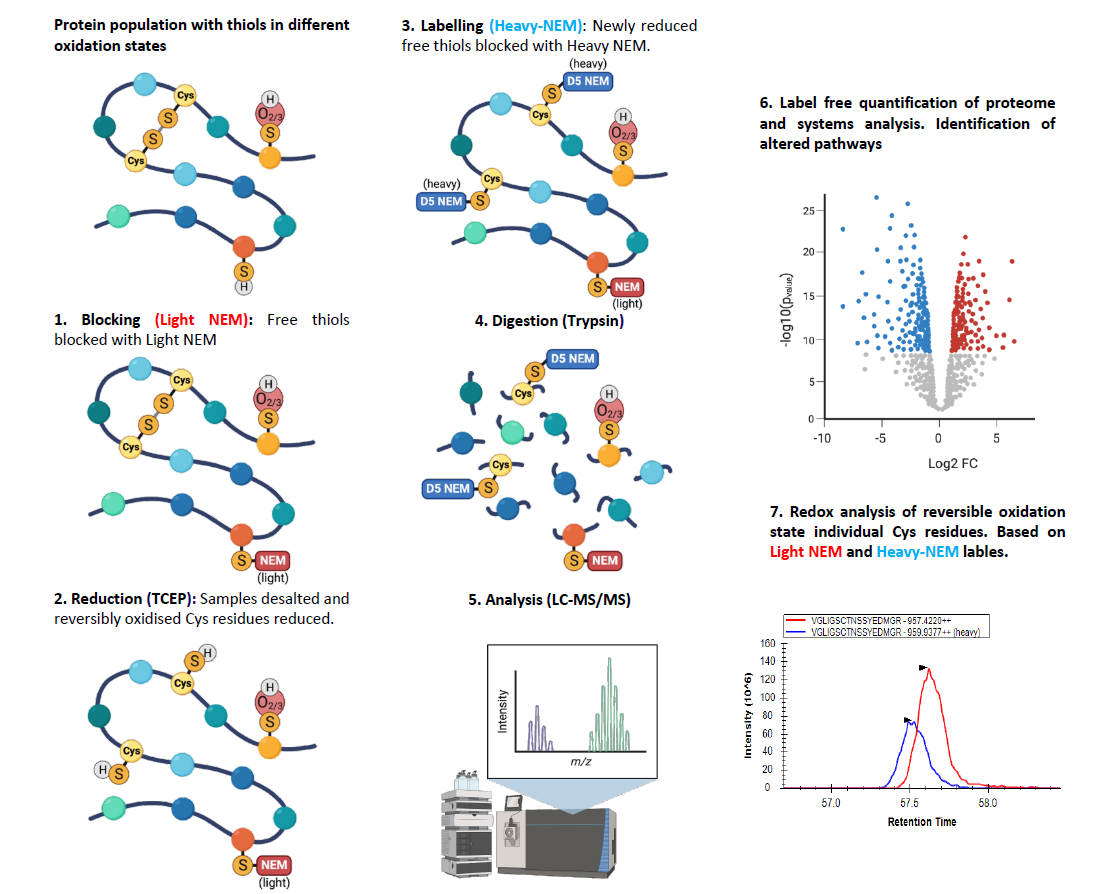


**Suppl. Table 1.**

| **Accession** | **Gene name** | **Peptide sequence** | **Cys** | **Log_2_FC Heavy NEM/ Light NEM** | | | | |
| --- | --- | --- | --- | --- | --- | --- | --- | --- |
|  |  |  |  | **N2 vs *prdx-2*** | **N2 vs *skn-1*** | **N2** | ***prdx-2*** | ***skn-1*** |
|  |  |  |  |  |  | **Ctrl vs Ex** | **Ctrl vs Ex** | **Ctrl vs Ex** |
| **B2D6P1** | **RMD-2** | **FCNEIGNRVEK** | **Cys43** | **-0.80** | **-2.03*** | **-2.26*** | **-0.26** | **0.47** |
| **C1P641** | **EPI-1** | **SGCPLHSVR** | **Cys3057** | **0.35** | **-0.48** | **0.30** | **0.56** | **1.81*** |
| **D0IMZ5** | **FLN-1** | **HQGSGHYVCSYR** | **Cys2225** | **1.65*** | **-0.28** | **-0.83** | **-1.62*** | **-3.91** |
| **G5ECA7** | **T02D1.8** | **SCMFGNQAIVDSFK** | **Cys48** | **-0.94** | **-4.90** | **-0.52** | **1.61*** | **4.59** |
| **G5EDZ9** | **CPI-1** | **QGSVQASQVTAANCPLK** | **Cys99** | **-0.80** | **-0.97** | **0.84** | **2.11*** | **1.90*** |
| **G5EE04** | **HIP-1** | **TDLATACK** | **Cys210** | **-0.38** | **0.14** | **1.61*** | **1.49*** | **0.31** |
| **G5EEK8** | **SCA-1** | **NCLFSGTNVASGK** | **Cys209** | **-0.85** | **-4.56** | **-2.18*** | **0.14** | **3.70** |
| **G5EF32** | **NPA-1** | **MLTVCGEVYK** | **Cys632** | **0.38** | **0.24** | **1.50*** | **1.34*** | **1.46*** |
| **G5EF32** | **NPA-1** | **AVVTGCK** | **Cys1415** | **0.57** | **1.89** | **1.52*** | **1.18*** | **-7.50** |
| **G5EF32** | **NPA-1** | **MLALCGEVYK** | **Cys881** | **1.02** | **0.74** | **1.98*** | **1.46** | **1.14** |
| **G5EGP8** | **CPZ-1** | **GPIACGIAATK** | **Cys217** | **2.62*** | **1.81*** | **3.08*** | **-7.90** | **0.42** |
| **O02286** | **PCK-1** | **FPGCMAGR** | **Cys164** | **-1.67*** | **-0.45** | **-1.03** | **0.74** | **-0.71** |
| **O02286** | **PCK-2** | **DEGWMAEHMLIMGVTRPCGR** | **Cys305** | **0.34** | **1.13** | **-0.73** | **0.53** | **-1.70*** |
| **O02640** | **MDH-2** | **NVQCAYVASDAVK** | **Cys278** | **-1.37*** | **-1.55*** | **-0.73** | **0.75** | **1.00** |
| **O17271** | **HEH-1** | **ADGCELTVK** | **Cys39** | **-0.18** | **0.78** | **2.40*** | **-3.49** | **0.28** |
| **O17725** | **D1086.3** | **CKPLEEDHK** | **Cys87** | **1.05*** | **0.21** | **1.27*** | **0.84** | **2.02*** |
| **O44451** | **PDHB-1** | **GPNGAAAGVAAQHSQDFSAWYAHCPGLK** | **Cys161** | **-1.07** | **0.18** | **1.57*** | **-0.99** | **-2.44** |
| **O44727** | **CPN-4** | **PISNFNCLENINQFSTAAR** | **Cys88** | **-1.85*** | **-1.75*** | **-1.53*** | **1.18*** | **0.18** |
| **O45815** | **ACT-5** | **FRCPEVLFQPAFIGMEGAGIHETTYQSIMK** | **Cys257** | **0.08** | **-1.31** | **-1.84*** | **-0.35** | **1.71** |
| **O62277** | **DCT-18** | **SENSFVPDQLCK** | **Cys121** | **1.37** | **0.65** | **1.78** | **0.52** | **2.50*** |
| **O76840** | **MIG-6** | **VQGPCSGK** | **Cys1513** | **-1.22** | **1.40** | **1.81*** | **3.34** | **1.18** |
| **O76840** | **MIG-6** | **DACHLNVDQGR** | **Cys1375** | **1.02** | **0.19** | **2.10*** | **0.88** | **2.75*** |
| **O76840** | **MIG-6** | **YTGCGGNANR** | **Cys1408** | **-0.25** | **-0.66** | **0.45** | **1.69*** | **1.69*** |
| **O76840** | **MIG-6** | **CDQLQPR** | **Cys1161** | **1.51** | **2.44*** | **1.49** | **2.31*** | **0.94** |
| **O76840** | **MIG-6** | **DTGPCTNFVTK** | **Cys1456** | **-0.64** | **-0.17** | **1.10** | **2.58*** | **2.12*** |
| **P05690** | **VIT-2** | **IRFTCLQR** | **Cys1571** | **0.24** | **0.29** | **1.75*** | **2.34** | **1.58*** |
| **P05690** | **VIT-2** | **YSSECPECEK** | **Cys228** | **-0.32** | **-0.36** | **1.37** | **2.53*** | **1.14** |
| **P05690** | **VIT-2** | **TLEGDCQVAYTVIR** | **Cys185** | **0.37** | **1.55*** | **0.14** | **-7.89** | **-0.67** |
| **P06125** | **VIT-5** | **APLTTCYSLVAK** | **Cys1330** | **-1.90*** | **-0.90** | **-0.51** | **-6.00** | **0.67** |
| **P06125** | **VIT-5** | **CITRPETAYGLR** | **Cys206** | **1.65*** | **1.09*** | **2.21*** | **-9.40** | **0.99** |
| **P06125** | **VIT-5** | **VCFSIEPVSECRR** | **Cys1533** | **0.56** | **0.33** | **-6.86** | **-6.92** | **1.88*** |
| **P06125** | **VIT-5** | **IRFTCMPR** | **Cys1560** | **0.81** | **-0.03** | **0.72** | **0.32** | **2.06*** |
| **P06125** | **VIT-5** | **VCFSIEPVSECR** | **Cys1533** | **0.84** | **0.07** | **1.11** | **-6.60** | **2.40*** |
| **P09446** | **HSP-1** | **MVNHFCAEFK** | **Cys243** | **-0.84** | **-1.38** | **0.60** | **2.56*** | **2.61*** |
| **P09446** | **HSP-1** | **ARFEELCADLFR** | **Cys307** | **-0.66** | **-1.56*** | **0.00** | **0.72** | **1.28*** |
| **P0DM42** | **ACT-3** | **FRCPEAMFQPSFLGMESAGIHETSYNSIMK** | **Cys258** | **-2.91*** | **-1.34*** | **-0.80** | **2.38*** | **0.85** |
| **P0DM42** | **ACT-3** | **CPEAMFQPSFLGMESAGIHETSYNSIMK** | **Cys258** | **-0.03** | **-1.44*** | **-1.30*** | **-0.33** | **1.71*** |
| **P11141** | **HSP-6** | **RTIEPCRK** | **cys245** | **-1.11** | **-1.86*** | **-0.55** | **1.43*** | **1.26*** |
| **P18948** | **VIT-6** | **CNYEREMFEK** | **Cys1514** | **1.31** | **0.29** | **2.14*** | **1.02** | **1.99*** |
| **P18948** | **VIT-6** | **QHEICFTQK** | **Cys1569** | **0.18** | **-0.09** | **1.24** | **1.46*** | **1.60*** |
| **P18948** | **VIT-6** | **NQQCGLCGHYDNEK** | **Cys1476** | **0.86** | **0.13** | **1.43** | **1.86** | **2.15*** |
| **P25807** | **CPR-1** | **TQWSECK** | **Cys98** | **2.29*** | **1.64*** | **1.28*** | **-1.23*** | **-1.09*** |
| **P27798** | **CRT-1** | **ADADLGDFHGETPYNVMFGPDICGPTRR** | **Cys133** | **1.63*** | **1.75*** | **1.87*** | **-7.74** | **0.73** |
| **P27798** | **CRT-1** | **ADADLGDFHGETPYNVMFGPDICGPTR** | **Cys133** | **1.37*** | **1.47*** | **2.42*** | **0.73** | **0.81** |
| **P27798** | **CRT-1** | **HEQGIDCGGGYVK** | **Cys101** | **0.50** | **0.65** | **1.02** | **2.57*** | **1.77*** |
| **P29691** | **EEF-2** | **LLEPVYLVEIQCPEAAVGGIYGVLNRR** | **Cys745** | **0.92** | **0.01** | **0.61** | **-1.61*** | **0.55** |
| **P29691** | **EEF-2** | **IWCFGPDGTGPNLLMDVTK** | **Cys645** | **0.30** | **-0.32** | **-0.55** | **-0.21** | **2.36*** |
| **P34455** | **ACO-2** | **TAVPSTIHCDHLIEAQK** | **Cys123** | **-0.25** | **-1.81*** | **0.09** | **0.59** | **0.83** |
| **P34686** | **CAP-2** | **RLPPQHCDK** | **Cys21** | **-0.74** | **-2.75** | **-5.07** | **1.87*** | **3.37** |
| **P34690** | **TBA-7** | **YMAVCLLYR** | **Cys314** | **-0.31** | **-1.58*** | **0.45** | **-0.21** | **1.07** |
| **P37165** | **UBL-1** | **ECQQPSCGGGVFMAQHANR** | **Cys120** | **-1.81*** | **-1.72*** | **-0.82** | **1.73*** | **0.54** |
| **P46561** | **ATP-2** | **VCLTGLTVAEYFR** | **Cys292** | **-0.82** | **-1.66*** | **-1.06** | **0.97** | **1.59*** |
| **P46769** | **RPS-0** | **LIDIGVPCNNK** | **Cys163** | **-2.59*** | **-1.13*** | **-0.28** | **2.14*** | **1.06*** |
| **P48154** | **RPS-1** | **IRSEMIGCIEK** | **Cys169** | **-0.62** | **-1.02** | **-1.70*** | **0.28** | **0.06** |
| **P48158** | **RPL-23** | **LNRLPSAGVGDMFVCSVK** | **Cys63** | **-1.46*** | **-1.85*** | **-3.93** | **0.71** | **0.89*** |
| **P49041** | **RPS-5** | **VNQAIWLLCTGAR** | **Cys161** | **-4.78** | **-1.60*** | **-0.25** | **0.41** | **-2.98** |
| **P50432** | **MEL-32** | **AEHVLDLAHIACNK** | **Cys439** | **-0.74** | **0.21** | **-0.03** | **1.69*** | **0.22** |
| **P50432** | **MEL-32** | **NTCPGDVSALRPGGIR** | **Cys414** | **-0.28** | **-0.44** | **-0.51** | **2.34*** | **-0.01** |
| **P52015** | **CYN-3** | **HTGPGVLSMANAGPNTNGSQFFLCTVK** | **Cys122** | **-0.20** | **0.06** | **-0.06** | **0.60** | **-2.69*** |
| **P52275** | **TBB-2** | **LTNPTYGDLNHLVSLTMSGVTTCLR** | **Cys239** | **-0.46** | **-1.31*** | **2.25*** | **-1.43*** | **1.12** |
| **P52275** | **MEC-7** | **TAVCDIPPR** | **Cys354** | **-0.54** | **-1.57*** | **-0.28** | **0.02** | **1.40** |
| **P55155** | **VIT-1** | **HLLNEASGSVCK** | **Cys1312** | **-5.45** | **1.68*** | **-5.53** | **0.41** | **-6.92** |
| **P55955** | **TTR-16** | **GVAVCNK** | **Cys32** | **-0.52** | **-1.55*** | **0.48** | **1.24*** | **2.18*** |
| **P90983** | **RPS-29** | **VCAGHHGLIR** | **Cys24** | **-2.98*** | **-2.20*** | **-1.55** | **3.99*** | **-0.86** |
| **P91020** | **C07D8.6** | **GCAILPK** | **Cys258** | **-0.18** | **-1.85*** | **-0.72** | **0.31** | **0.48** |
| **P91423** | **T03F1.11** | **DDVICMLLGAEK** | **Cys30** | **-1.48*** | **-1.66*** | **-0.49** | **0.46** | **0.60** |
| **P92005** | **CPZ-2** | **IQGLVEEGCNVYR** | **Cys313** | **1.32*** | **1.55*** | **1.00*** | **0.97** | **-0.57** |
| **Q03577** | **DRS-1** | **NCFLVLR** | **Cys100** | **-1.04** | **-1.26*** | **-1.00** | **1.57*** | **0.32** |
| **Q09533** | **RPL-10** | **MLSCAGADRLQTGMR** | **Cys105** | **-1.32** | **-1.67*** | **-1.74*** | **-0.07** | **0.45** |
| **Q10454** | **F46H5.3** | **SLQGYPFNPCLSEANYLEMESK** | **Cys179** | **-1.56*** | **-0.86** | **-1.31** | **1.20*** | **1.25** |
| **Q10454** | **F46H5.3** | **LGWLTFCPSNLGTTVR** | **Cys312** | **-0.64** | **-1.64*** | **-0.74** | **0.36** | **0.65** |
| **Q10576** | **DPY-18** | **HAACPVLVGIK** | **cys496** | **-0.22** | **-2.05*** | **0.59** | **-0.58** | **0.87** |
| **Q10663** | **ICL-1** | **LAADVCGVPTIIVAR** | **Cys218** | **-2.20*** | **-2.34*** | **-1.13** | **1.69*** | **1.22** |
| **Q10663** | **ICL-1** | **DSTAIQYCIDR** | **cys271** | **-1.73** | **-2.08*** | **-0.22** | **0.18** | **-1.30** |
| **Q17473** | **TTR-18** | **VTCNGQPAENIK** | **Cys34** | **1.26** | **-0.12** | **1.57*** | **0.50** | **1.65*** |
| **Q17994** | **GOT-2.2** | **NWEHITNQIGMFCFTGINPQQVEK** | **Cys366** | **-2.11** | **-0.22** | **1.56*** | **0.41** | **-1.67** |
| **Q18786** | **SNR-4** | **NNHQVLINCR** | **Cys46** | **-0.22** | **0.17** | **-0.78** | **-0.78** | **-1.76*** |
| **Q18787** | **RPT-1** | **LCPNSTGAEIR** | **Cys379** | **-2.51*** | **-1.08*** | **-1.55*** | **0.99** | **-5.45** |
| **Q19162** | **RPL-11** | **IAVHCTVRGPK** | **Cys79** | **-1.13** | **0.68** | **0.18** | **1.84*** | **-0.72** |
| **Q19591** | **NSPG-10** | **TVMPAQAGWFGCQSAR** | **Cys201** | **2.16*** | **-5.99** | **1.99** | **0.55** | **0.33** |
| **Q19626** | **VHA-12** | **IALTSAEFLAYQCK** | **Cys269** | **-1.77*** | **-2.34*** | **-1.52*** | **1.70*** | **0.71** |
| **Q19842** | **PCCA-1** | **HIEMQVLCDK** | **Cys262** | **-1.27** | **-2.08*** | **-2.14*** | **-0.78** | **0.01** |
| **Q20206** | **RPS-11** | **KCPWAGNVPIR** | **Cys57** | **-1.49*** | **-2.82** | **-2.06*** | **0.96** | **0.06** |
| **Q20206** | **RPS-11** | **DIHPGDLVTIGECRPLSK** | **Cys128** | **-0.76** | **-0.81** | **-0.37** | **1.58*** | **0.81** |
| **Q20970** | **MRS-1** | **LVVVLCNLK** | **Cys816** | **0.49** | **-1.70*** | **-0.72** | **-0.29** | **1.33*** |
| **Q21824** | **PRDX-3** | **HGEVCPADWHEDSPTIK** | **Cys199** | **-0.25** | **-2.06*** | **0.87** | **-0.06** | **0.77** |
| **Q22054** | **RPS-16** | **TATAVAHCK** | **Cys23** | **-1.90** | **0.08** | **-2.04*** | **2.25** | **-0.46** |
| **Q22285** | **TTR-46** | **LLCGNGPAANVR** | **cys31** | **-2.23** | **-2.03*** | **0.69** | **1.08** | **3.56** |
| **Q22494** | **VHA-15** | **LACFGTTR** | **Cys154** | **-1.13** | **-1.55*** | **-0.69** | **0.17** | **0.52** |
| **Q22562** | **T19B10.2** | **CVLAANDLSHNAWDK** | **Cys76** | **0.92** | **0.57** | **1.69*** | **-5.67** | **-5.51** |
| **Q22633** | **HPD-1** | **PCQDRPTLFLEIIQR** | **Cys341** | **1.50*** | **-0.22** | **-0.43** | **-1.56*** | **0.07** |
| **Q23120** | **RSP-2** | **YSRPCSTR** | **Cys108** | **-0.17** | **0.11** | **2.65*** | **0.25** | **-3.21** |
| **Q23258** | **ZC373.2** | **TVVSAPIDGAPGTATCSDTTTPER** | **Cys44** | **0.62** | **0.45** | **1.42*** | **1.71*** | **1.22** |
| **Q23258** | **ZC373.2** | **LACGFSCSR** | **Cys21** | **0.76** | **-0.19** | **-7.11** | **-0.15** | **2.13*** |
| **Q23621** | **GDH-1** | **VIGIQEYDCAVYNPDGIHPK** | **Cys313** | **-1.84*** | **-0.57** | **-0.34** | **1.81*** | **0.08** |
| **Q27527** | **ENOL-1** | **SCNCLLLK** | **Cys338** | **-0.49** | **-1.50*** | **-1.08** | **0.36** | **0.48** |
| **Q2EEM8** | **TTR-45** | **LLCGTSPAK** | **Cys30** | **0.60** | **1.42*** | **1.93*** | **1.08** | **0.22** |
| **Q5FC40** | **D1086.10** | **LEDLTNYCGQR** | **Cys152** | **0.66** | **0.22** | **1.67*** | **0.34** | **1.22** |
| **Q8MXS8** | **Y47G6A.22** | **VNAVCPGVTVTNLHR** | **Cys181** | **-2.09*** | **-2.02*** | **-4.82** | **-2.24** | **1.52** |
| **Q93934** | **ADK-1** | **SLCAHLAAANTFTQDHLQK** | **Cys134** | **-1.47*** | **-1.75*** | **-1.34*** | **1.01** | **0.49** |
| **Q94246** | **GFI-1** | **CIDPTVVPPVYPVK** | **Cys312** | **2.47*** | **-6.55** | **-6.51** | **0.34** | **0.33** |
| **Q95XJ0** | **Y69A2AR.18** | **GLCGGVHSSIVK** | **Cys107** | **-1.01** | **-1.75*** | **-1.78*** | **0.26** | **0.08** |
| **Q95Y04** | **RPS-28** | **TGSQGQCTQVRVEFINDQNNR** | **Cys22** | **-3.03*** | **-2.34*** | **-2.37*** | **-1.00** | **-1.44** |
| **Q9BKU5** | **Y37E3.8** | **AGGACVLVA** | **Cys141** | **-2.01*** | **-1.55*** | **-1.21*** | **1.15** | **0.13** |
| **Q9BL34** | **COX-6B** | **QCFAYYVDFHR** | **Cys66** | **0.68** | **0.53** | **1.62*** | **1.52*** | **1.50** |
| **Q9BL34** | **COX-6B** | **DFCPGFWTERWDELLSEGRFPAK** | **Cys98** | **0.29** | **0.36** | **1.62** | **1.88*** | **-0.21** |
| **Q9N4Y8** | **NUO-5** | **VLVDPGMTILQACALVGVDIPR** | **Cys53** | **-1.74*** | **-1.78*** | **-1.39*** | **1.22** | **-0.21** |
| **Q9TXU7** | **EIF-1.A** | **VQVFCFDGK** | **Cys51** | **-0.35** | **-0.58** | **1.75*** | **0.09** | **0.10** |
| **Q9TZS5** | **CCT-7** | **AQACTLLLR** | **Cys368** | **-1.23*** | **-1.71*** | **-1.10*** | **0.60** | **0.86** |
| **Q9U2A8** | **RPL-43** | **ATGIWNCAK** | **Cys57** | **-0.92** | **-1.53*** | **-0.37** | **0.72** | **1.20*** |
| **Q9XWU9** | **Y37D8A.19** | **CTDAASNPAER** | **Cys54** | **0.50** | **-0.71** | **1.01** | **1.88*** | **2.43*** |

**Suppl Table 2**

| Figure | Strains | Condition | # Deaths/ # Total | Mean lifespan | Log-rank test, *p*-value |
| --- | --- | --- | --- | --- | --- |
| 4a | N2 | Control | 101/105 | 13.85 ± 0.38 | compared to Control |
|  | *bli-3(e767)* |  | 97/105 | 10.28 ± 0.29 | < 0.0001 |
|  | *prdx-2(gk169)* |  | 99/105 | 11.08 ± 0.35 | < 0.0001 |
|  | *skn-1(zj15)* |  | 99/105 | 10.78 ± 0.35 | < 0.0001 |
| 4c | N2 | Control | 99/105 | 12.05 ± 0.37 |  |
|  |  | Exercise | 100/105 | 13.22 ± 0.4 | 0.0357 |
|  | *prdx-2(gk169)* | Control | 96/105 | 10.86 ± 0.36 |  |
|  |  | Exercise | 95/105 | 9.81 ± 0.29 | 0.0071 |
|  | *skn-1(zj15)* | Control | 97/105 | 10.67 ± 0.33 |  |
|  |  | Exercise | 97/105 | 10.42 ± 0.31 | 0.4003 |
| 4d | N2 | Control | 75/75 | 2.84 ± 0.22 |  |
|  |  | Exercise | 75/75 | 4.39 ± 0.30 | < 0.0001 |
|  | *prdx-2(gk169)* | Control | 75/75 | 3.35 ± 0.27 |  |
|  |  | Exercise | 75/75 | 2.51 ± 0.19 | 0.0112 |
|  | *skn-1(zj15)* | Control | 75/75 | 2.67 ± 0.21 |  |
|  |  | Exercise | 75/75 | 2.31 ± 0.17 | 0.1592 |
| 4e | N2 | Control | 80/80 | 10.45 ± 1.05 |  |
|  |  | Exercise | 80/80 | 15.70 ± 1.33 | 0.0033 |
|  | *prdx-2(gk169)* | Control | 80/80 | 16.33 ± 1.48 |  |
|  |  | Exercise | 80/80 | 5.5 ± 0.54 | < 0.0001 |
|  | *skn-1(zj15)* | Control | 80/80 | 10.35 ± 0.94 |  |
|  |  | Exercise | 80/80 | 8.53 ± 0.84 | 0.0886 |

| Mean lifespan was determined by OASIS2 platform, Statistical analysis was performed using GraphPad Prism 7. |
| --- |
| Number of worms represents the number of dead worms scored relative to total number of worms initially started with. The difference in number is indicative of censored worms. |
